# Mandatory fortification with folic acid for the prevention of neural tube defects: a case study of Australia and New Zealand

**DOI:** 10.1007/s00381-022-05823-x

**Published:** 2023-01-19

**Authors:** Lyall Thurston, Barry Borman, Carol Bower

**Affiliations:** 1 Rotorua, New Zealand; 2 Environmental Health Intelligence NZ and the NZ Congenital Anomalies Register, Massey University-Wellington, Wellington, New Zealand; 3 Telethon Kids Institute, Nedlands, Australia; 4 University of WA, Nedlands, Western Australia Australia

**Keywords:** Neural tube defects, Folic acid, Mandatory fortification, Consumer advocacy, Austalia, New Zealand

## Abstract

**Purpose:**

To present a case study of the considerations of mandatory fortification with folic acid in Australia and New Zealand.

**Methods:**

Review of published reports and consumer advocacy views.

**Results:**

Australia and New Zealand jointly approved mandatory fortification of flour with folic acid to prevent neural tube defects in 2007. Fortification was fully implemented in Australia in 2009 and has resulted in reduction in NTD. At the last minute, industry lobbying led to the New Zealand government not proceeding with fortification. With continued consumer advocacy, mounting scientific evidence, and a change of government, approval was given in 2021 for mandatory fortification of flour with folic acid.

**Conclusion:**

In large part as a response to consumer pressure, New Zealand has now joined with Australia (and around 70 other countries) in fortifying flour with folic acid for the prevention of NTD.

## Background

Based on comprehensive case ascertainment of neural tube defects (NTD) from state-based birth defects registers in Australia, the total prevalence of NTD diagnosed in terminations of pregnancy, live births, and stillbirths in the 1980s and early 1990s was around 2 per 1000 births [1–4]. The prevalence in New Zealand over a similar time period was slightly lower, but data sources were thought to be less complete [4].

Many observational studies between the 1960s and the late 1980s supported the role of folic acid in reducing NTD, prompting several randomised controlled trials to be undertaken. Since the publication, in the early 1990s, of evidence from these trials for the prevention of neural tube defects with folic acid [5, 6], public health recommendations were made in many countries, including Australia and New Zealand [7, 8]. These recommendations included the use of periconceptional folic acid supplements and fortification of staple foods such as bread and cereals with folic acid.

In line with these recommendations, campaigns to promote the use of periconceptional folic acid supplementation were undertaken in Australia, resulting in 30–50% of Australian women taking periconceptional folic acid supplements [9–14]. Although there were no national health promotion campaigns in New Zealand, by 2011, most New Zealand women had heard of the need for folic acid supplements, 14% of women deliberately consumed food in the periconceptional period due to its folate content,and one-third of women consumed folic acid supplements prior to conception [15].

A 30% fall in the prevalence of NTD was documented [16] following promotional activities. However, the trials had shown that a 70% reduction in NTD was possible, and there was evidence of disparities in reach of these health promotion approaches, such that younger, less educated women were less aware of the need to take periconceptional folic acid, and those who were smokers or had unplanned pregnancy were less likely to have taken folic acid supplements. [11, 15].

Even when pregnancy is planned, periconceptional supplement use is less common among Māori, Pacific, and Asian women, younger women, and women with lower education and income in New Zealand [15]. NTD show a similar socioeconomic gradient and a higher risk among Māori women than those of non-Māori non-Pacific ethnicity in New Zealand [15].

In Australia, Aboriginal infants had a higher prevalence of NTD prior to folate promotion, and the promotional activities did not result in any change, further widening the gap between Aboriginal and non-Aboriginal NTD prevalence [17, 18].

At the same time as health promotion campaigns were underway, and as a means of reaching all women to boost their folate intake prior to and in early pregnancy, fortification of staple foods with folic was being considered by the governments of Australia and New Zealand. This was undertaken through their joint agency, Food Standards Australia New Zealand (FSANZ).

## Food Standards Australia New Zealand (FSANZ)

The overarching food policy in Australia and New Zealand is set by government ministers with responsibility for food regulation, who make up the Australian and New Zealand Ministerial Forum on Food Regulation. This Forum develops food regulatory policy and policy guidelines, to which FSANZ must have regard when setting food standards. Once standards are set in the Australia New Zealand Food Standards Code by FSANZ, the Forum can amend, adopt, or reject them and can ask FSANZ to review them or make new ones (www.foodstandards.gov.au). Once adopted, the code is enforced by states and territories in Australia and by the Ministry for Primary Industry in New Zealand; FSANZ has no enforcement powers.

## Fortification of food with folic acid in Australia and New Zealand

### Voluntary fortification

Unlike in many other countries, folic acid was not an allowable additive to foods in Australia and New Zealand prior to 1995. An expert panel was convened in 1994 to consider fortification with folic acid, resulting in permission to voluntarily add folic acid to certain foods in 1995 in Australia and 1996 in New Zealand. An evaluation of voluntary folic acid fortification in 2001 [19] found that relatively few of the recommended foods had been fortified (mainly breakfast cereals), few women of childbearing age knew which foods were fortified, and there was no evidence that voluntary fortification had reduced neural tube defects in Australia or New Zealand [20].

### Mandatory fortification

Following the report on evaluation of voluntary fortification, FSANZ was asked to consider mandatory fortification (among other options). Over the ensuing 6 years, FSANZ produced, in accordance with their processes, an initial assessment report, a draft report, a final report and a first review report [16]. Consumers, industry, health professionals, and government agencies made many submissions. Special assessments were commissioned on the risks and benefits of increased folate intake, including on cancer, cardiovascular disease, twinning, cognitive function, vitamin B12 deficiency, and the potential effect of incremental increases in folic acid intake on NTD in Australia and New Zealand [21]. Along with an assessment of feasibility and cost, FSANZ concluded, in 2006, that mandatory fortification of flour for bread-making with folic acid was the preferred approach in Australia and New Zealand. And the Food Regulation Ministerial Council confirmed, in 2007, the mandatory fortification of wheat flour for bread-making with 200–300 ug folic acid per 100 g of flour. Mandatory folic acid fortification was implemented in Australia in September 2009.

## Monitoring and outcomes in Australia

A comprehensive monitoring framework was established [22, 23]. Three examples are presented here, illustrating the effectiveness of mandatory fortification.

1. A very early retrospective analysis of serum and red cell folate levels in over 20,000 blood samples collected for other reasons at a large public hospital in New South Wales from 2007 to 2010 found a reduction in low serum and red cell folate levels following the introduction of mandatory fortification [24].
2. In a research project in Western Australia, folate status was estimated among Aboriginal people before and after fortification: dietary folate intake was assessed, and red cell folate was measured. Prior to fortification, 10% of the samples in women and 26% in men were folate deficient, whereas 2 years after fortification, no participant had red cell folate deficiency, and the mean red cell folates had increased by over 40%. There had been no change in use of folic acid supplements (low (< 7%) before and after fortification), there was continued high use of shop-bought bread, and there was a significant increase in dietary folate equivalents consumed daily, with over 140 DFE coming from fortified bread in the post-fortification era [25].
3. Trends in NTD following mandatory fortification have been examined using data from five Australian states and territories [26]: an overall 14.4% reduction in NTD was observed, compared with the pre-fortification period (the proportion expected based on estimates done prior to mandatory fortification) [21]. For Aboriginal births in this analysis, there was a 75% reduction in NTD.

## What happened in New Zealand?

Industry resistance to mandatory fortification was strong in both countries, and ultimately, their lobbying in New Zealand carried sufficient sway to overcome the solid science, the careful considerations of risks and benefits, the submissions from consumers, industry and health, and the accumulating evidence from overseas, of the effectiveness of mandatory fortification in preventing NTD. At the end of this lengthy and thorough process, the New Zealand government chose not to proceed with mandatory fortification.

The major change in approach came with the election of a new government in New Zealand in 2008. On 27 August 2009, Kate Wilkinson, the Minister for Food Safety in the new national government, halted the process to mandatory fortification. Although she agreed that folic acid can prevent NTD, she insisted there needed to be greater consumer choice, and the baking industry was prepared to developed a voluntary code to increase women’s intake of folic acid. In 2009, the New Zealand Food Safety Agency (NZFSA) undertook the fourth public consultation on the options for folic acid fortification (previous public consultations were in 2004, 2006, and 2007) [27–29].

In March 2014, the New Zealand Association of Bakers (NZAB) agreed to a Code of Practice with an aspirational goal of fortifying a minimum of 25% and up to 50%, by volume, reported as production volume numbers, of packaged sliced loaf breads (including private label products) marketed and distributed in New Zealand, by their members. The 2017 annual report on the “Voluntary Fortification of Bread with Folic Acid” found that 38.0% by production volume, of packaged sliced bread marketed or distributed by NZAB members or their private label partners, was fortified with folic acid by 2017 [30].

Strong advocacy continued for and against mandatory fortification. In 2017, the Ministry of Health requested the Office of the Prime Minister’s Chief Science Advisor and the Royal Society Te Apārangi to review the health benefits and risks of folic acid fortification of food. The conclusion was that the benefits of mandatory fortification of packaged bread with folic acid outweigh any potential adverse effects [31].

A consumer study commissioned by the Ministry for Primary Industries (MPI), in 2017, found that more than half of respondents supported voluntary fortification, and just under a quarter favouring a mandatory programme [32].

Subsequently, the MPI conducted a further public consultation in October–November 2019 [33]. As a consequence of 106 submissions, the majority supporting mandatory foritifcation, and the recommendation of MPI, on 8 July 2021, the government announced approval to add folic acid to non-organic wheat flour for bread-making.

Folate Fortification – the New Zealand Story: Advocacy Perspective In the late 1980s, New Zealand’s greatest child health researcher and children’s advocate, the late Professor Sir Bob Elliott, established the Folate Replenishment-Plus Committee at the University of Auckland’s School of Medicine where he was the Foundation Professor of Paediatrics. The committee included academics, paediatricians, parents of children with spina bifida, epidemiologists, milling and baking representatives, and Rotarians. Substantial funding was provided to the committee by Rotary, and Dr. Seema Singh, a graduate working with Professor Sir Bob Elliott, was the lead researcher.

The call for mandatory fortification met with a demand and challenge from politicians that all political parties would have to commit. With intense lobbying and countless meetings with politicians from all sides of the New Zealand House of Parliament, this was achieved in January 2001.

Over ensuing years, lobbying continued, and numerous approaches were made to government to have folic acid included in flour—a common staple. The government proposed implementing voluntary fortification which pro-fortification lobbyists, quoting international science and research, believed was setting the proposal up to fail.

Industry remained firm in its opposition. Industry was ambivalent and saw little commercial value in adding folic acid to flour as a public health initiative. Industry’s repeated response to government over the decades was for government to legislate mandatory fortification not to request the milling and baking industry to commit to voluntary fortification.

New Zealand and Australia have a joint food standard, and the issue became a major topic on their agenda, promoted at the time by the New Zealand Minister for Food Safety, the Hon. Dame Annette King (currently the New Zealand High Commissioner to Australia). In June 2007, the mandatory folic fortification standard was accepted by the Food Safety Ministers of Australia and New Zealand with a 2-year implementation period to commence in both Australia and New Zealand in 2009. Coincidentally, in the late 2008, New Zealand had a general election and a change of government. Industry lobbied the new centre-right government who were considered industry “friendly” to scuttle the introduction of mandatory fortification.

The new centre-right government under pressure from industry in the first days in office in May 2009 withdrew New Zealand from the joint agreement with Australia. Australia subsequently proceeded alone with mandatory fortification, and New Zealand reverted to a voluntary regime. The New Zealand government maintained it was offering consumers’ choice. A minister at the time is on record stating that the change in policy was based on consumer choice not science!

Since 2009, lobbying has been ongoing to have mandatory fortification introduced in New Zealand.

On 12 October 2010, the New Zealand Parliament voted 77 votes to 44 to disallow mandatory fortification of bread with folic acid in New Zealand.

In 2012, after an 8-week consultation process, the government chose to keep the inclusion of folic acid voluntary. The report also acknowledged that mandatory fortification was associated with lower rates of NTDs. A study in 2018 found that less than half of packaged bread had folate added.

Finally, in July 2021, the Hon. Dr Ayesha Verrall, the Minister of Food Safety, announced that mandatory fortification of non-organic bread flour would be mandated to take effect in 2 years. New Zealand would effectively join around 70 countries worldwide who have mandatory fortification with folic acid as a public health initiative.

## Conclusions

Firm evidence of the prevention of NTD with folic acid was published in the early 1990s, and the Centers for Disease Control and Prevention in the USA were quick to propose mandatory fortification of flour, and it was instituted in 1998. In Australia and New Zealand, the process was very much slower, at times cumbersome, and frequently frustrating for those of us who could see the benefits unfolding overseas and were concerned that Australian and New Zealand families were not being provided with the best means to prevent these serious birth defects. Nevertheless, it was a thorough process, and the final result included the voices of consumers and industry as well as health professionals and policy makers. It was therefore astonishing that, at the last minute, political will overturned this sound decision-making process in New Zealand. Campaigners for mandatory fortification there were devastated, but not destroyed. They continued their advocacy and championing of the science, seeking every opportunity to ensure the issue did not fall off the agenda, and the final decision is in large part due to their unerring faith in its importance. Twelve years after mandatory fortification of flour with folic acid was instituted in Australia, New Zealand joined its trans-Tasman neighbour in mandating folic acid fortification.

## Data Availability

The authors declare that the data supporting the findings of this study are available within the article and its references.
